# 3D Antenna Characterization for WPT Applications

**DOI:** 10.3390/s21134461

**Published:** 2021-06-29

**Authors:** Marina Jordão, Diogo Pires, Daniel Belo, Pedro Pinho, Nuno Borges Carvalho

**Affiliations:** 1 Instituto de Telecomunicações, Departamento de Eletrónica, Telecomunicações e Informática, Universidade de Aveiro, Campus Universitário de Santiago, 3810-193 Aveiro, Portugal; drppires@ua.pt (D.P.); a35842@ua.pt (D.B.); nbcarvalho@ua.pt (N.B.C.); 2 Instituto de Telecomunicações, 3810-193 Aveiro, Portugal; ptpinho@av.it.pt; 3 ISEL—Instituto Superior de Engenharia de Lisboa, Instituto Politécnico de Lisboa, 1959-007 Lisboa, Portugal

**Keywords:** 3D antenna array, Internet of Things (IoT), multi-sines, over-the-air (OTA), wireless power transmission (WPT), wireless sensor network (WSN)

## Abstract

The main goal of this paper is to present a three-dimensional (3D) antenna array to improve the performance of wireless power transmission (WPT) systems, as well as its characterization with over-the-air (OTA) multi-sine techniques. The 3D antenna consists of 15 antenna elements attached to an alternative 3D structure, allowing energy to be transmitted to all azimuth directions at different elevation angles without moving. The OTA multi-sine characterization technique was first utilized to identify issues in antenna arrays. However, in this work, the technique is used to identify which elements of the 3D antenna should operate to transmit the energy in a specific direction. Besides, the 3D antenna design description and its characterization are performed to authenticate its operation. Since 3D antennas are an advantage in WPT applications, the antenna is evaluated in a real WPT scenario to power an RF–DC converter, and experimental results are presented.

## 1. Introduction

Fifth generation (5G) of communications brings optimization and modernization of today’s infrastructures. Internet of Things (IoT) networks will be boosted by the 5G network implementation. Consequently, people and objects will communicate and their information must be shared easily [1]. Moreover, the use of wireless sensor networks (WSNs) will increase significantly and the use of sensors will be massive, which leads to a huge challenge in terms of power supply. Therefore, since more sensors are used, more power is then required to power all those devices and, as a consequence, more batteries will be required. Thus, solutions to reduce and/or eliminate batteries from sensors are a current hot topic.

Wireless power transmission (WPT) and energy harvesting are arising as an alternative to batteries, since these techniques aim to power devices without using batteries [2] and wires. For these reasons, batteryless sensors have been selected for several applications and, for instance, RF–DC converters are being used to power devices in WPT applications. The RF–DC converters are nonlinear devices, where the DC component is obtained since the device rectifies a time varying signal [3]. To achieve higher efficiency, the input power should be increased in the RF–DC converters. Nevertheless, to obtain the input signal of the RF–DC converters, the input port of this device should be connected to an antenna.

WPT systems require a transmitting antenna to transmit the signal to the RF–DC converter, and the selection of this antenna should be done carefully in order to achieve more efficient transmission. Moreover, in literature, there are several works that demonstrated that multi-antennas can improve the WPT systems’ efficiency. For instance, in [4], a beam-steering antenna is presented to power up wireless sensors, where the beam is controlled to obtain high directivity. By using this strategy, the space dispersion by the environment can be compensated and higher power is available to the wireless sensor antenna, typically allowing it to operate at a higher power conversion efficiency. In [5], a transmitting antenna array is used to power a batteryless device. In this case, the active antenna elements were controlled in three different states in order to deliver constant power to the device, considering a scenario where the WPT sensor is moving. In [6], it was analyzed, that by using a 2D transmitting multi-antenna and a receiver 2D multi-antenna, the RF WPT systems’ efficiency can be increased. The 2D transmitting multi-antenna is composed of 64 elements and the receiver multi-antenna is formed by 16 elements, and simulations and experiments were performed to validate this experiment. In [7], random deployment of a non-collocated transmitting multi-antenna composed of seven identical antennas was used to demonstrate that, by using an OTA multi-sine calibration method in those antennae, the RF–DC converters used in WPT systems can be optimized to achieve higher efficiency. The authors of [8] presented a WPT system which used a 2D transmitting multi-antenna composed of 64 elements and a receiver multi-antenna composed of 16 elements, where a WPT algorithm was designed to improve the transmission performance. This work presents higher efficiency results when compared with multi-antenna with random phase schemes.

However, other types of antennas can be selected to power batteryless sensors. Three-dimensional (3D) antenna arrays are being explored for several purposes, for instance, for satellite, train, ship, and boat communications [9,10,11,12,13]. These type of antennas present the benefit of radiating in several directions with a constant gain, which is ideal to power WPT sensors scattered over a wide area around it by using beam switching techniques. The transmitted signal can be sent through all the azimuth directions and reach distinct elevation angles without mechanical moving. Considering an indoor scenario, where RF–DC converters are placed in different locations to power different types of IoT devices, a 3D antenna may be the best choice to transmit the power signals to the devices.

In this way, in [10], a 3D antenna is presented in an icosahedron-truncated form. The near-spherical shape provides the antenna’s gain stabilization, down to very small elevation angles. In addition, the authors in [9] demonstrate that a 3D antenna with hemispherical coverage can be used to reach a specific region because, using only a single antenna element, some areas cannot be covered. The patterns produced by the single elements individually combining with the ones obtained by the combination of two consecutive active elements ensures the coverage of the certain hemispherical region.

A 3D antenna array composed of six patch antennas for indoor position estimation systems is discussed in [14]. The antenna can locate the target by estimating both the azimuth theta and elevation phi direction of arrival of the incoming signals. For simultaneously wireless information and power transfer (SWIPT) applications, in [15], a 3D antenna array composed of eight patch antennas in a low-cost structure is presented. The antenna structure was built in a 3D printer and the antenna presents equal antenna gain over the azimuth plane, which aims to equalize the effective radiated power (ERP) over all directions. Regarding localization applications, a low-cost circularly polarized pentagonal patch-excited sectorized antenna is presented in [16], with a robust semi-spherical coverage. Moreover, in [17], a 3D compact antenna with six ports is used for RF energy harvesting systems, demonstrating that this type of antenna is suitable for IoT applications. Furthermore, the authors suggested that the rectifier circuit can be used in this scenario to power IoT devices by being excited by this 3D antenna. Thus, an advantage of using 3D antennas instead of 2D antennas in WPT systems is regarding coverage. For instance, if a WPT system is placed in an indoor environment (room) to power several devices, using a 3D antenna in the middle of the room allows radiating in any room direction. On the contrary, the 2D antennas have a limited radiation zone.

In summary, all these antennas have in common the capability of radiating in several directions, where radiation pattern, the directivity, and, in some cases, the tracking performance with all the elements operating are validated with simulations and experimental results. Although, in terms of tracking performance, the contribution of each antenna element is not analyzed when all elements are operating simultaneously or when beam-switching is performed. Thus, the overall performance of the antenna, through all the azimuth directions, cannot be accurately retrieved. 

Nevertheless, to have beam steering capabilities, the design of 3D antennas is quite complex due to the nonconventional array factor. Therefore, the beam-switching approach in 3D antennas becomes an interesting solution in order to reduce complexity. With this in mind, since 3D antennas have several elements in different positions and with beam-switching capabilities, the overall antenna characterization becomes a complex task. An efficient and low-cost strategy to select the proper 3D antenna element to perform beam-switching in each scenario is a requirement. 

Over-the-air (OTA) characterization is arising, since it is an interesting and simple solution to test several types of 5G systems. In [18], an OTA characterization method is proposed using multi-sine. The method was applied in [19] to identify malfunction in multiple input multiple output (MIMO) antennas, where a specific measurement system was developed together. However, this method can be applied not only to identify malfunction in antenna arrays with several elements, but also for other purposes. In [20], this OTA multi-sine method was used to calibrate the transmitting MIMO antennas’ main beam to be aligned with the receiver. 

This leads to the main goal of this work, which is to present a 3D antenna capable of performing robust coverage for WPT systems in all directions and its characterization. In addition to the typical characterization procedures to validate antennas, the OTA multi-sine method will be used to perform the characterization of the 3D antenna to identify which element should be activated in order to transmit the signal for a specific direction. By using this strategy, the contribution of each element to reach a specific direction can be evaluated. Moreover, an RF–DC converter will be designed and used to demonstrate the effectiveness of the 3D antenna to power the batteryless device. 

This manuscript is divided as follows. The 3D antenna array is described in Section 2. In Section 3, the OTA multi-sine technique is explained in detail. The 3D antenna array characterization is performed in Section 4. The application of the 3D antenna array in a WPT system is presented in Section 5. Finally, in Section 6, conclusions are drawn.

## 2. 3D Antenna Array

3D antenna arrays have the capability of radiating in several directions, which is a benefit for several applications, as mentioned previously. In order to achieve robust coverage across 360° azimuth angles and at different elevation angles, the 3D antenna arrays must be carefully designed. The number of antenna elements should be enough and be strategically positioned to reach all the intended directions.

**Figure 1:**
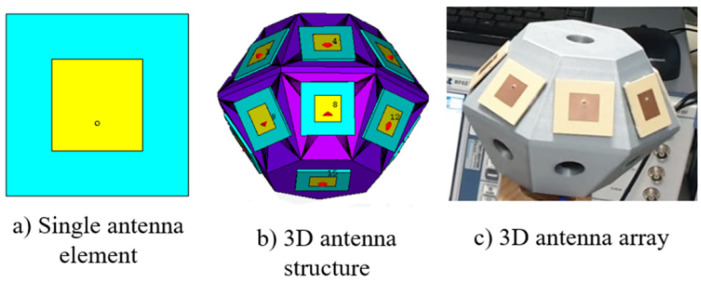
3D antenna array design.

Since microstrip antennas present a simple implementation, a flexible design, and are low-cost, these antennas were selected for this work. They were built on Isola Astra substrate (ϵr = 3, tanδ = 0.0017, and h = 0.762) and designed to operate at 5.55 GHz. The antenna elements are separated by 0.71 λ_0_. The single antenna element design is presented in Figure 1a.

The 3D antenna array with 15 elements was designed, as shown in Figure 1b. The antenna array was designed to have 14 antenna elements in the side faces, equally divided through two circular bands (seven in each circular band), in order to radiate over all the hemisphere and to different elevation angles. One element is placed in the bottom to cover the above plane. Since the antenna elements are relatively close to each other (0.71λ_0_), the combination of two consecutive elements produced a beam for a specific direction. The 3D structure was designed using SolidWorks software and printed in polylactic acid (PLA) (dielectric constant ϵr = 2.7 and dissipation factor, tg(δ) = 0.008 @ 1 MHz) and is presented in Figure 1c. 

Moreover, the main goal of using this antenna is to power passive or low-power sensors in WPT scenarios. Consequently, the 3D antenna array should follow a beam-switching operation; in other words, all the antenna elements should not be simultaneously active. This means that, to control the antenna radiation pattern, switching between active antenna elements should be performed. For that matter, when the single antenna elements are individually active, it is important to investigate the radiation patterns, as well as when two consecutive elements are simultaneously active. In addition, since antenna elements are relatively close to each other, mutual coupling between elements occurs when two active antenna elements are operating, and this should be taken into account.

## 3. OTA Multi-Sine Technique

Antennas composed of several elements present a challenge in terms of characterization, which is the case with 3D antenna arrays. Since this antenna type has several elements, strategies to identify problems in each element are relevant because several elements will be operating. As mentioned previously, in [18,19], the authors present a strategy to identify malfunctions in active antenna arrays OTA. The same technique is used in [20] to calibrate MIMO antenna arrays OTA. Nevertheless, in this work, the same technique will be applied, but to identify which elements of the 3D antenna should operate to produce a beam in a specific direction. 

**Figure 2:**
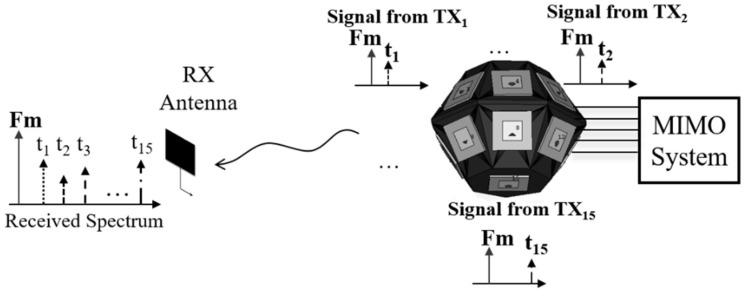
OTA multi-sine technique.

With this in mind, the applied OTA technique uses multi-sines to identify each antenna element, as is illustrated in Figure 2. 

As can be seen, each antenna element is fed with two tones: the main tone (*F_c_*) and a tickle tone (*t_i_*). The main tone is equal in all antenna elements. On the other hand, the tickle tone of each antenna element operates at different frequencies in order to associate each antenna element with a different tickle tone. 

Moreover, the tickle tones have a small amplitude compared with the main tone. By using an MIMO system composed of several transmitters, each antenna element can be fed by each transmitter with a main tone and a tickle tone. Then, a receiver antenna is connected to a receiver to acquire the resulting spectrum with all contributions of the antenna elements. In this way, the information regarding each transmitter antenna element can be obtained to evaluate if each element is operated properly. Since this work aims to present and characterize a 3D antenna array for WPT applications, this technique highlights the importance of understanding which antenna elements should be operating to produce a beam in a specific direction.

## 4. 3D Antenna Array Characterization

Since the 3D antenna array is composed of several elements with the main goal of performing beam-switching, several characterization steps were performed as follows.

### 4.1. Antenna Element Characterization

**Figure 3:**
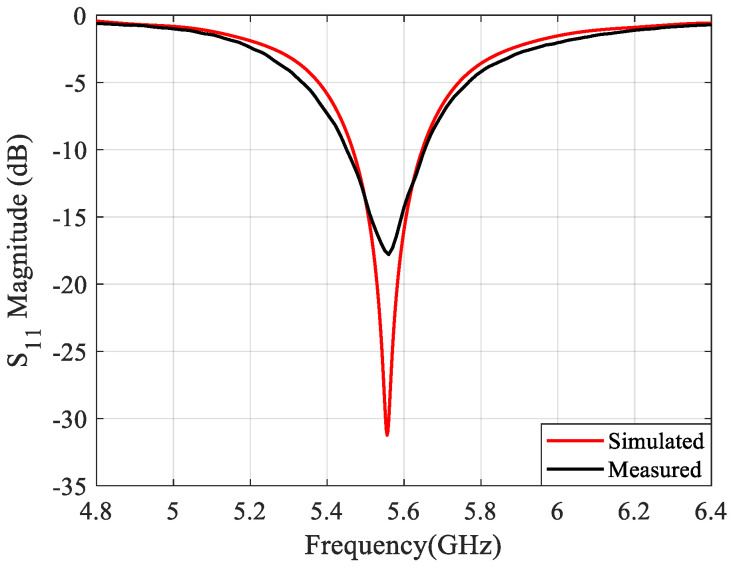
Microstrip antenna element reflection coefficient magnitude, simulated and measured.

During the process design of the microstrip antenna, electromagnetic (EM) simulations were performed using computer science technology (CST) software in order to validate their operation. In Figure 3, the magnitude of the simulated reflection coefficient (S11) of the antenna element can be seen. 

The gain achieved by simulation for this antenna element is 6.73 dBi. Moreover, this antenna element was characterized using a vector network analyzer (VNA), and the resulting measured S11 is shown in Figure 3 as well. In addition, it can be seen that the measured reflection coefficient is matched to 50 ohm for the intended operating frequency, 5.55 GHz, presenting a reflection coefficient magnitude of −17.51 dB.

### 4.2. Mutual Coupling Simulations

**Figure 4:**
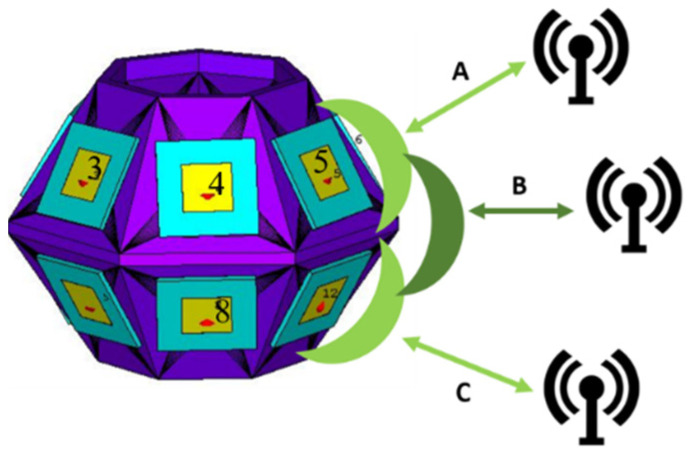
3D antenna array beam-switching possibilities example.

The mutual coupling between elements should be evaluated, since one of the goals of using this 3D antenna is to perform beam-switching. With this in mind, the active reflection coefficient (ARC) should be analyzed. This coefficient is obtained when the antenna ports are excited simultaneously [21]. The ARC is the reflection coefficient for a single antenna element in the presence of mutual coupling. Therefore, simulations were performed to evaluate the ARC for two consecutive elements simultaneously active, since only two consecutive elements will be operating to perform beam-switching. Figure 4 illustrates an example of the possibilities to perform beam-switching around one element and the respective antenna elements. 

As an example, in this figure the antenna element 4 is analyzed, and its neighbor elements. As can be seen in Figure 4, the antenna element 3 is the element to the left of element 4, element 5 is the element to the right of element 4, and element 8 is the element below the element. These elements were selected because they represent the possible combinations in the 3D antenna array to perform beam-switching and, consequently, the mutual coupling introduced by the antenna elements is analyzed. 

**Figure 5:**
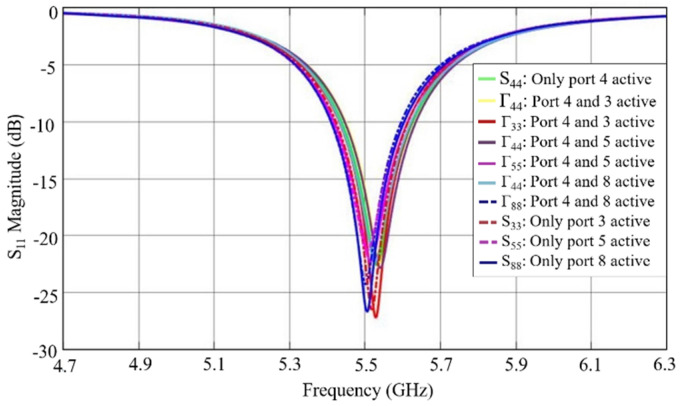
Simulations to evaluate the mutual coupling between 3D antenna elements.

Figure 5 shows the ARC simulations, as well as the magnitude of the reflection coefficient of those 3D antenna elements. 

S_xx_ represents the reflection coefficient magnitude that occurs when only this port is active (others are not operating), while Γ_xx_ represents the ARC magnitude of the port when the neighbors’ elements are operating. From those results, it can be concluded that the antenna matching for the intended frequency (5.55 GHz) is validated, since the mutual coupling results that occur between elements can be neglected.

### 4.3. Radiation Patterns Simulations

As mentioned previously, Figure 4 demonstrated an example of the possibilities to perform beam-switching, where three scenarios are considered in order to ensure a hemispherical coverage. When two elements are selected, a higher gain is guaranteed, but with a narrower radiation beam. However, there are directions along 360° which benefit from the two active elements (the zones between the main lobes). 

In this sense, in case A, the target is considered to be placed above the 3D antenna array and two elements of the upper band are selected. In case C, the target is considered to be placed below the 3D antenna array and two elements of the lower band are selected. When the target is at the level of the antenna array, two elements are selected, one element from the upper band and one element from the lower band (the one right below it), which is illustrated by case B. 

**Figure 6:**
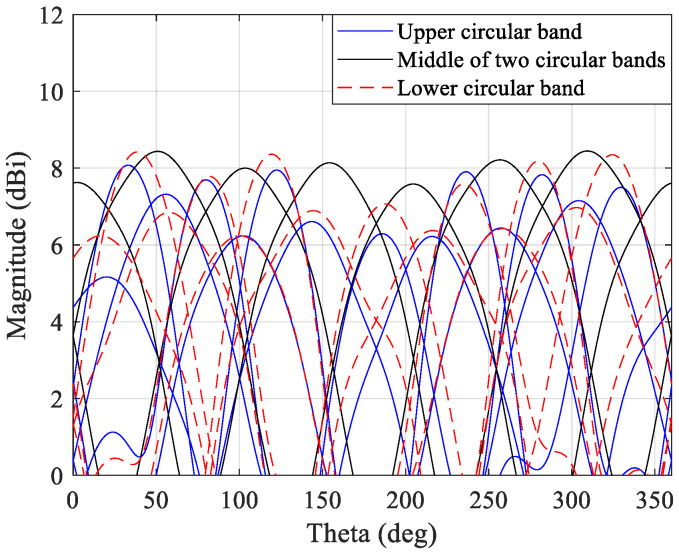
Different radiation pattern obtained by the 3D antenna array.

Figure 6 shows the simulation results for the radiation patterns achieved by the 3D antenna array for the three different scenarios, using CST. In Figure 6, case A is identified by the upper circular band simulations, case C is illustrated by the lower circular band, and case B is represented by the middle of two circular bands.

From those simulations, a slight antenna gain variation over the azimuth plane is observed. This is due to the fact that the antenna elements are not placed in the 3D structure perfectly, but there are some small differences. These small differences may lead to modifying of the antenna element input impedance and attenuate some of these elements. In the maximum radiation intensity directions, the gain is approximately 8.1 dBi, but small gaps occur between the main patterns and these zones, with a gain of approximately 7 dBi. Comparing these simulation results, it can be concluded that the signal produced by the elements of both circular bands (case B) is the one that presents better gain stabilization, although the upper and lower bands present similar behavior.

### 4.4. OTA Multi-Sine Characterization

To analyze the 3D antenna array radiation capabilities, the OTA multi-sine technique was used. By using the main tone, at the same frequency in all antenna elements, and a tickle, with small power in relation to the main tone and at a different frequency in all antenna elements, the identification of each antenna element can be performed. Thus, by using the tickle tone information, a specific direction can be achieved. Despite the intended goal of using the 3D antenna array not being to have all the antenna elements operating at the same time (simultaneously activated), the OTA multi-sine characterization aims to obtain the exact azimuth angles achieved by each antenna element, which makes the characterization process quicker and more efficient. 

**Figure 7:**
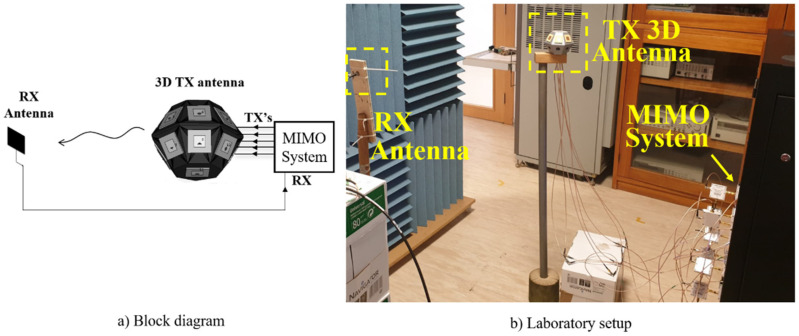
OTA multi-sine characterization to identify the contribution of each antenna element for case A (upper band.): (**a**) block diagram and (**b**) laboratorial setup.

With this in mind, from [22], a specific setup was utilized to perform the OTA multi-sine characterization of the 3D antenna array. The block diagram and the laboratory setup are shown in Figure 7, and consist of three elements: an MIMO system, a transmitter 3D antenna array, and a receiver antenna. The transmitting 3D antenna was previously described above. The receiver antenna is a single antenna patch equal to the 3D antenna elements, operating at 5.55 GHz, as illustrated in Figure 2. The MIMO system is composed of Universal Software Radio Peripheral (USRP)s, with several transmitters and receivers. Thus, each 3D antenna array element is excited by one transmitter with a main and a tickle tone (multi-sine). Then, the receiver patch antenna obtains the signals from the 3D antenna, and it is connected to a receiver to process the information.

The main goal is to excite the 3D antenna array with the multi-sine OTA, and the receiver antenna will perform a full azimuth coverage and obtain the multi-sine information. For this reason, the bottom antenna element will not be considered. Since the transmitter 3D antenna is composed of two bands, the upper and the lower bands, each one with seven elements, seven transmitters will be connected to the 3D antenna elements. Then, for the seven elements (first, seven for the upper band, and, then, seven for the lower band), several tickle tone frequencies were selected, which are presented in Table 1. 

These frequencies were selected in order to be as close as possible to the main tone. As illustrated in Figure 2, the tickle tones are generated with small power in relation to the main tone and, in this experiment, the tickle tones were generated with a power difference of −20 dBm from the main tone. All the antenna elements were excited in phase. 

**Figure 8:**
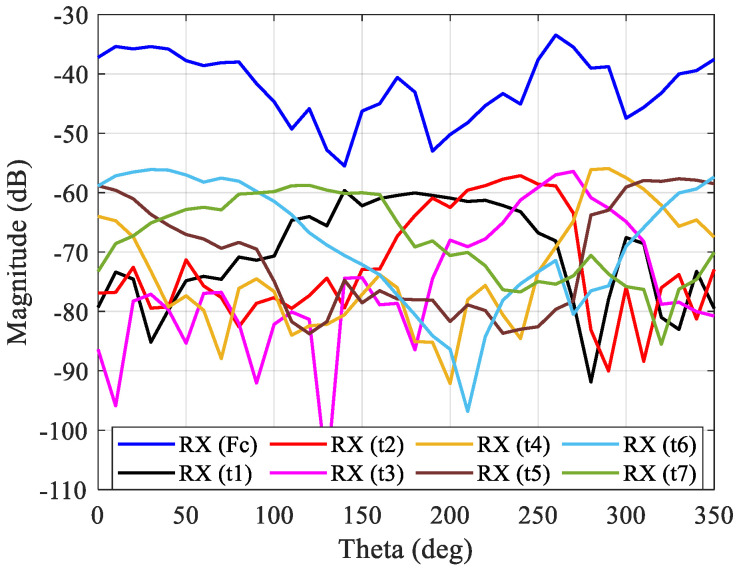
OTA multi-sine characterization to identify the contribution of each antenna element for case A (upper band).

The characterization was performed for two different elevation angles, in order to simulate two distinct cases: case A to evaluate the upper band of the antenna, and case B to evaluate the lower band of the antenna, as described previously in Figure 4. In these experiments, the receiving antenna was placed in several positions around the transmitting 3D antenna (which is fixed) to obtain the signal from the main tone and tickle tones between 0° and 350°, with a step of 10°. For case A, the transmitting 3D antenna was placed at 1 m high, and the receiver antenna was placed at 1.3 m to evaluate the upper band. The corresponding results are presented in Figure 8. 

**Figure 9:**
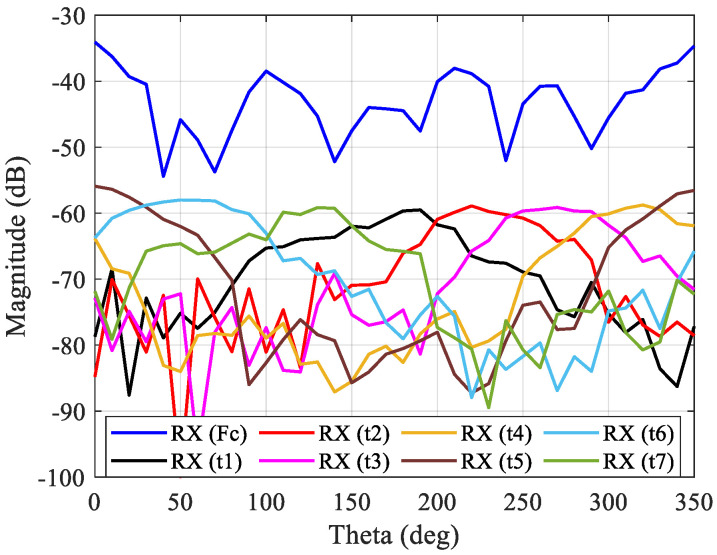
OTA multi-sine characterization to identify the contribution of each antenna element for case C (lower band).

For case C, the transmitting 3D antenna was placed at 1 m high and the receiver antenna was placed at 0.7 m to evaluate the lower band, and the corresponding results are presented in Figure 9.

For both cases, since the main tone was generated with more power, this one presents a higher power in relation to the tickle tones as expected. Moreover, the main tone presents several oscillations over the 360 azimuth degrees, which occurs due to the multipath and fading effects in the laboratory environment, since the measurements were not performed in one anechoic chamber. In addition, the receiver antenna rotation movement around the transmitter 3D antenna was subject to slight errors because the measurements were performed manually, which causes a different signal path. Concerning the tickle tones, they provide information regarding the radiation pattern of each antenna element in each azimuth direction, as can be seen in Figure 8 and Figure 9. For specific azimuth direction, the overlapping of tickle tones occurs, which aims to identify the azimuth angles where they present the highest radiation intensity. As a consequence, by using the tickle tones information to perform switching or not, the activation of the proper antenna elements can be performed in order to transmit the energy through the azimuth plane and at different elevation angles.

## 5. WPT Application

The 3D antenna arrays present the capability of radiating in several directions with a constant gain, and this is a benefit for WPT applications because WPT sensors can be powered using this antenna. Thus, in order to demonstrate this, a WPT system was projected where an RF–DC converter circuit was powered by the transmitting 3D antenna.

### 5.1. RF–DC Converter Circuit

Typically, an RF–DC converter is composed of different blocks: an antenna, a matching network, a nonlinear rectifying device, a low-pass filter, and a resistive load. Several configurations have been presented in the literature to convert RF into DC signal [23], where semiconductors are used as rectifier elements. These devices are selected for several application scenarios due to them being low cost and small in size. These characteristics are relevant, since the WPT technique operates based on EM radiation. For this reason, it is expected that converters are excited by a low power signal, due to the different types of attenuation, such as free space path losses and multi-path effects. 

Usually, diodes are selected as rectifier elements in RF–DC converter circuits, although, some converters operate with transistors in a diode configuration. More than one rectifier element can be used in these circuits, which impacts the conversion efficiency and the available output power of the device. The *Schottky* diodes are being used as nonlinear rectifiers due to their smaller depletion zone, which leads to a smaller junction capacity and, consequently, lower conduction voltage and switching speed [24,25]. Therefore, in accordance with the requirements to achieve, the RF–DC converter configuration should be chosen carefully. 

**Figure 10:**
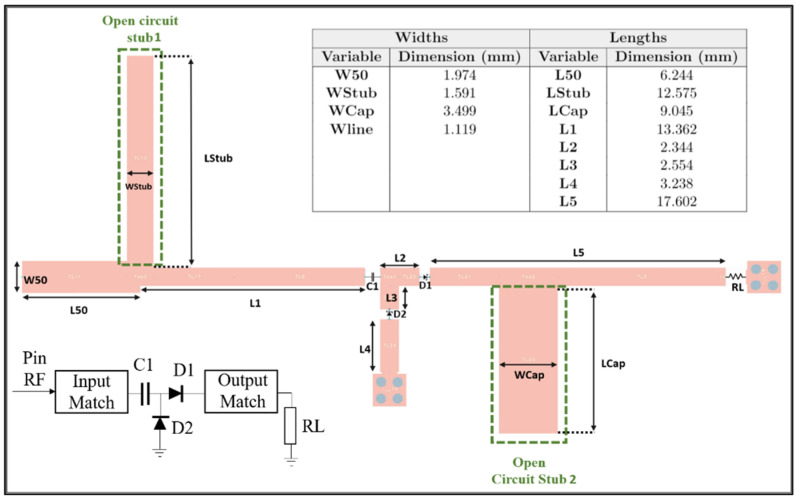
Schematic of the RF–DC converter.

Since the main goal of this transmitter 3D antenna array is to be applied in WPT scenarios, the RF–DC converter circuit design should take into account two essential requirements. First, the device should achieve high efficiency, and, second, it must be projected to produce considerable output DC voltage in order to power WPT sensors. With this in mind, ensuring the perfect match between the antenna and the converter to avoid power losses and maximize the overall conversion efficiency is the first step. The converter should be composed of more than one nonlinear rectifier to produce considerable output DC voltage. Nevertheless, by using several nonlinear rectifiers, the efficiency decreases due to the resistance associated with a fully polarized diode and more losses will be introduced in the signal path. Considering these facts, the design of the RF–DC converter used in this WPT system followed the single-stage multiplier topology [26], as shown in Figure 10.

The *Schottky* diodes SMS7630-079LF were selected as nonlinear rectifiers to design the RF–DC converter. Moreover, the matching network is based on an open-circuit stub in parallel with a series line, as shown in Figure 10. A load resistance (RL) of 2.6 kΩ was used. The RF–DC converter was designed and optimized to operate at 5.55 GHz and for an input power of 0 dBm.

**Figure 11:**
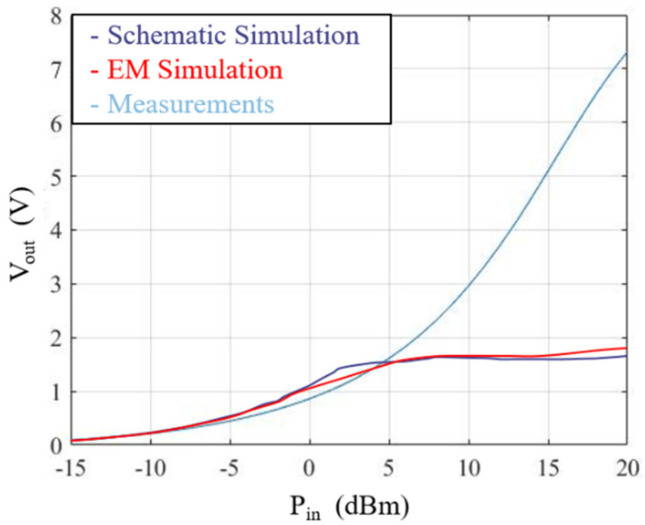
Simulated RF–DC converter circuit DC output voltage results.

In order to validate the converter design, schematic and EM simulations were performed, using an advanced design system (ADS), and compared. The output DC voltage simulations for several input powers are demonstrated in Figure 11 and it can be seen that schematic and EM simulations are very similar. 

**Figure 12:**
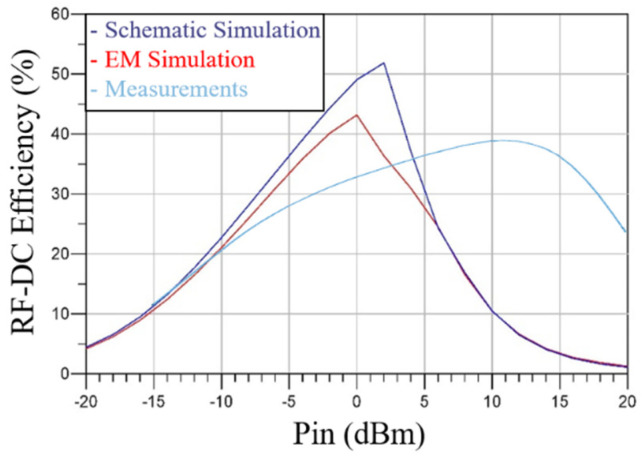
Simulated RF–DC converter circuit efficiency results.

Consequently, Figure 12 shows the RF–DC converter efficiency for several input powers. 

**Figure 13:**
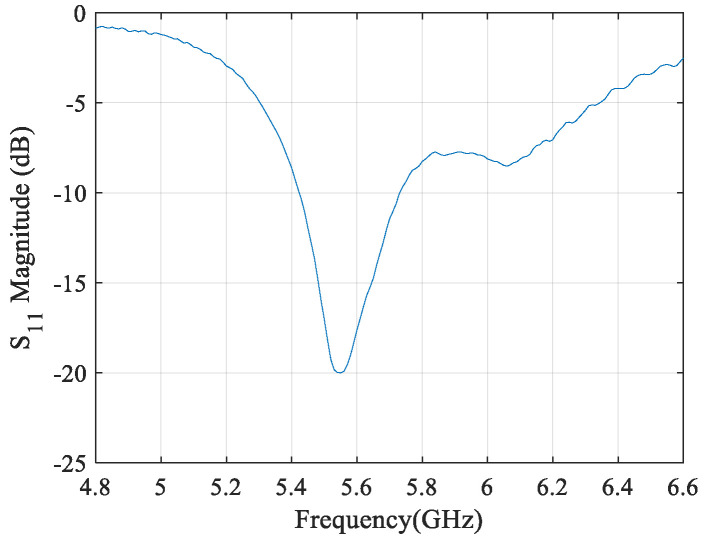
RF–DC converter circuit reflection coefficient magnitude measured on VNA.

Additionally, the RF–DC converter was characterized using VNA to evaluate the circuit reflection coefficient, and the results are depicted in Figure 13. From those results, it can be concluded that, for the intended frequency, 5.55 GHz, the RF–DC converter is matched to 50 Ohm, presenting the reflection coefficient magnitude of −20 dB. 

To evaluate the efficiency of the RF–DC converter, a specific setup was utilized. A vector signal generator (VSG) is used to generate an RF signal and is connected to the RF port of the RF–DC converter. The DC port of the converter is attached to a voltmeter to obtain the correspondent DC voltage. 

Similar to simulations, two different measurements were carried out. First, for several input power values, the RF–DC converter DC output voltage was measured, and the results are shown in Figure 11. Then, the RF–DC efficiency for several input powers was obtained, and the results are shown in Figure 12. The differences between the simulation results and the measurement results are justified by the fact that the diode model is far from the diode breakdown.

### 5.2. WPT System

**Figure 14:**
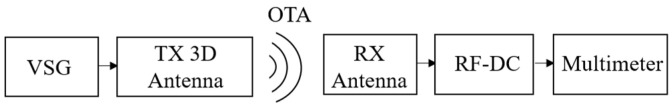
Block diagram of the measurement setup to characterize the WPT system.

The WPT system is composed of several elements: the transmitting 3D antenna, a VSG, the receiver antenna, the RF–DC converter, and a voltmeter. The VSG connected to the transmitting 3D antenna array will generate the RF signal. A receiver antenna is connected to the RF input port of the RF–DC converter circuit to obtain the signal from the transmitting antenna. A voltmeter is connected to the DC output port of the RF–DC converter to measure the resulting voltage generated by the converter. The block diagram used to characterize the WPT system is presented in Figure 14. 

**Figure 15:**
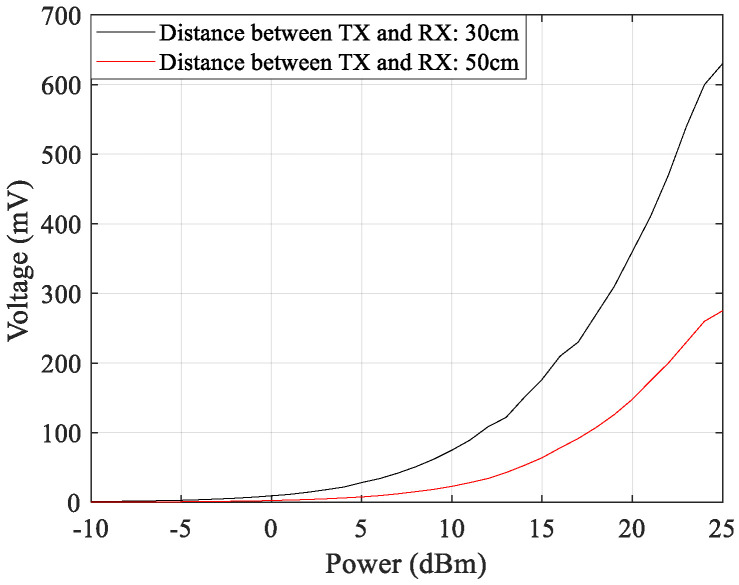
WPT system DC output power for two different distances and distinct transmitted powers.

Several experiments were performed. In the first experiment, for two different distances, the output DC voltage generated by the converter was measured as a function of the transmitted powers. The receiver antenna and the RF–DC converter circuit were placed at a distance of 30 cm and 50 cm, respectively, and the corresponding results are shown in Figure 15. 

**Figure 16:**
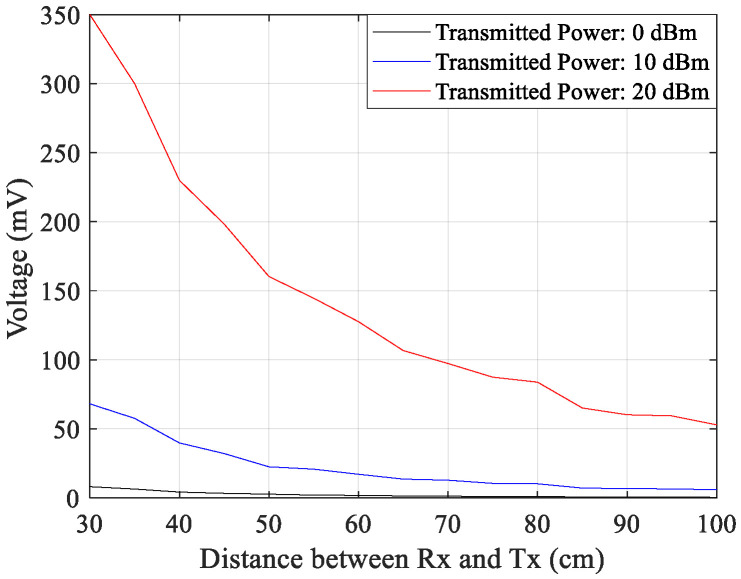
WPT system DC output power for different distances and distinct transmitted powers.

A second experiment was performed for different transmitted power as a function of the distances between the transmitting 3D antennas and the receiver antenna. The results are presented in Figure 16. By analyzing the results, it can be seen that the WPT system, using the transmitting 3D antenna for higher power values, is capable of powering passive or low-power sensors.

## 6. Conclusions

A new and alternative design of a 3D antenna array was proposed to improve WPT systems, with the main goal of powering sensors. Moreover, a detailed characterization process of the 3D antenna was performed, where the OTA multi-sine characterization technique was first applied to characterize and identify the antenna elements to carry out beam-switching. Consequently, the application of this technique allowed the detection of the contribution of each antenna element to reach a specific direction, which is an interesting solution to understand and characterize the individual operation of each antenna element.

In addition, the transmitted 3D antenna was applied in a WPT, where an RF–DC converter was designed for this goal, and it was demonstrated that 3D transmitting is an interesting solution to power sensors. Consequently, considering WPT systems where RF–DC converters are selected to power sensors, the 3D antenna can be an interesting solution for indoor WPT scenarios. With this solution, sensors can circulate in the entire indoor environment without any concern in relation to powering them, because the coverage provided by the 3D antenna is almost uniform in all directions.

## Figures and Tables

**Table 1:** Multi-sines frequencies used in each port of the 3D antenna array.

Fc	5.55 GHz
t1	Fc+112 kHz
t2	Fc+117 kHz
t3	Fc+122 kHz
t4	Fc+127 kHz
t5	Fc+132 kHz
t6	Fc+137 kHz
t7	Fc+142 kHz

## Data Availability

Not applicable.
